# Race/ethnicity and HAART initiation in a military HIV infected cohort

**DOI:** 10.1186/1742-6405-11-10

**Published:** 2014-01-24

**Authors:** Erica N Johnson, Mollie P Roediger, Michael L Landrum, Nancy F Crum-Cianflone, Amy C Weintrob, Anuradha Ganesan, Jason F Okulicz, Grace E Macalino, Brian K Agan

**Affiliations:** 1 Department of Preventive Medicine and Biometrics, Infectious Disease Clinical Research Program, Uniformed Services University of the Health Sciences, Bethesda, MD, USA; 2 Infectious Disease Service, San Antonio Military Medical Center, San Antonio, TX, USA; 3 Biostatistics Division, University of Minnesota, Minnesota, MN, USA; 4 Naval Health Research Center, San Diego, CA, USA; 5 Infectious Disease Clinic, Walter Reed National Military Medical Center, Bethesda, MD, USA

**Keywords:** HIV, HAART, Race, Ethnicity, Indications for HIV treatment, Disparities in care, African Americans

## Abstract

**Background:**

Prior studies have suggested that HAART initiation may vary by race/ethnicity. Utilizing the U.S. military healthcare system, which minimizes confounding from healthcare access, we analyzed whether timing of HAART initiation and the appropriate initiation of primary prophylaxis among those at high risk for pneumocystis pneumonia (PCP) varies by race/ethnicity.

**Methods:**

Participants in the U.S. Military HIV Natural History Study from 1998-2009 who had not initiated HAART before 1998 and who, based on DHHS guidelines, had a definite indication for HAART (CD4 <200, AIDS event or severe symptoms; Group A), an indication to consider HAART (including CD4 <350; Group B) or electively started HAART (CD4 >350; Group C) were analyzed for factors associated with HAART initiation. In a secondary analysis, participants were also evaluated for factors associated with starting primary PCP prophylaxis within four months of a CD4 count <200 cells/mm^3^. Multiple logistic regression was used to compare those who started vs. delayed therapy; comparisons were expressed as odds ratios (OR).

**Results:**

1262 participants were evaluated in the analysis of HAART initiation (A = 208, B = 637, C = 479 [62 participants were evaluated in both Groups A and B]; 94% male, 46% African American, 40% Caucasian). Race/ethnicity was not associated with HAART initiation in Groups A or B. In Group C, African American race/ethnicity was associated with lower odds of initiating HAART (OR 0.49, p = 0.04). Race and ethnicity were also not associated with the initiation of primary PCP prophylaxis among the 408 participants who were at risk.

**Conclusions:**

No disparities in the initiation of HAART or primary PCP prophylaxis according to race/ethnicity were seen among those with an indication for therapy. Among those electively initiating HAART at the highest CD4 cell counts, African American race/ethnicity was associated with decreased odds of starting. This suggests that free healthcare can potentially overcome some of the observed disparities in HIV care, but that unmeasured factors may contribute to differences in elective care decisions.

## Introduction

HIV has become a treatable illness in the era of highly active antiretroviral therapy (HAART), and HAART is associated with a reduction in morbidity and mortality among those with severe immunosuppression [1]. There is increasing evidence that HAART is associated with reduced mortality among those initiating HAART at higher CD4 cell counts as well [2-7]. Thus the optimal timing of HAART initiation may result in a more robust immunologic and virologic response, which may improve outcomes. However there are factors other than the degree of immune suppression involved in the decision to start HAART.

Despite the known benefits of HAART, certain groups appear to be more vulnerable to treatment disparities, and these disparities have persisted over time. In a U.S. study of HIV-infected persons, delays of three or more months between diagnosis and an initial specialty encounter occurred more frequently among patients who lacked access to care at diagnosis and among those of African American or Latino race/ethnicity [8]. Delays in the initiation of HAART and failure to receive PCP prophylaxis occurred more frequently among blacks or Latinos, women, and the underinsured compared with other HIV patients [9]. Among a national sample of patients eligible to receive HAART by published guidelines, African American race/ethnicity, female gender and injection drug use were all associated with decreased likelihood of receipt of HAART [10]. Finally, in patients with HIV treated at multiple clinical sites in the United Kingdom, black race was associated with starting antiretroviral therapy at a lower CD4 count [11].

The etiology of these disparities is complex and likely multifactorial. Factors that have been hypothesized from previous studies include lack of health insurance or other barriers impeding healthcare access, variations in patient education or health literacy, and aspects of the physician-provider relationship [12,13]. Understanding the role these factors play in creating barriers to optimal care is needed and may offer an explanation for some of these observed differences in HIV care and outcomes [14-17].

In this study, we evaluated factors associated with HAART initiation within a defined time window in a United States military cohort of persons with HIV. As a secondary analysis, we also analyzed factors associated with the initiation of primary pneumocystis pneumonia (PCP) prophylaxis among persons at highest risk for PCP. Our population consists of an ethnically diverse group of early-diagnosed HIV patients with free healthcare and medications [18]. Furthermore, since military service for those on active duty provides a stable source of income and members have a minimum high school education, this cohort may offer somewhat less confounding due to socioeconomic status. Time windows to evaluate HAART initiation were established based on military policy requiring that HIV infected active duty members with stable disease undergo medical evaluation every six months and standard practice to evaluate those with advanced HIV disease more frequently, at least every three to four months. HAART initiation was assessed in accordance with the U.S. Department of Health and Human Services (DHHS) guidelines for the use of antiretroviral agents [19], which, since 1998, have been adopted into the practice of many U.S. physicians who specialize in HIV care, including those practicing within the U.S. military system.

## Methods

### Study population

The U.S. Military HIV Natural History Study (NHS), a cohort study of military beneficiaries with HIV infection including active duty personnel, retirees and dependents, enrolled 5261 participants from 1986 to 2009. Active duty personnel must be HIV negative prior to entry into U.S. military service, and subsequently undergo HIV screening every one to five years. Service members and beneficiaries identified with HIV are referred to military treatment facilities (MTFs) across the U.S. for evaluation and treatment. Those participating in the study provide written informed consent prior to assuming a schedule of evaluations by an HIV specialist at least every six months at one of five participating MTFs. Data including demographic characteristics, medical history, and laboratory measurements are collected at each study visit as previously described [18]. In compliance with the Helsinki Declaration, the NHS protocol has been reviewed and approved by the institutional review boards (IRBs) centrally and at the study sites. All subjects provided informed consent for participation. This analysis was reviewed and approved by the central IRB.

Participants who were enrolled in the NHS from 1998-2009 and who had not initiated HAART before 1998 were included in this substudy. Participants were excluded from these analyses if they had died (N = 1195), were lost-to-follow-up (N = 969), or had initiated HAART prior to January 1, 1998 (N = 1514). The analysis also excluded those participants who, prior to the three-month window preceding the first study date, i.e. September 1, 1997, had a CD4 cell count below 200 cells/mm^3^, an AIDS-defining condition, or any severe symptoms (category B symptoms according to the 1993 Revised Classification System for HIV Infection, including refractory mucosal candidiasis, wasting syndrome, pre-invasive cervical cancer, bacillary angiomatosis, oral hairy leukoplakia, and chronic diarrhea) [20] (N = 233).

### Study design

Participants were followed from the later of January 1, 1998 or their date of initial diagnosis with HIV until meeting criteria for assignment in one of three groups based on concurrent DHHS criteria. The details of this classification as well as changes in DHHS criteria during the study period of 1998-2009 are summarized in Table 1. The three groups included:

• Group A – those participants with a definite indication for HAART. Participants were classified as “initiators” if they started HAART within four months of classification or “delayers” if they did not. Those with less than four months of follow-up between the date of classification and last study visit were excluded from analysis (N = 18).

• Group B – those participants with an indication to consider HAART. Initiators started HAART within six months of classification and delayers did not start HAART within this timeframe. Those with less than six months of follow-up between the date of classification and last study visit were excluded from analysis (N = 77).

• Group C – those participants who may have electively started HAART; initiators started HAART prior to being categorized into either Groups A or B; delayers were HAART-naive as of their last study visit and had not yet been categorized into Groups A or B.

**Table 1:** **Study group classifications according to changes in the DHHS guidelines for initiation of antiretroviral therapy in chronically**-**infected HIV patients**^**1**^

**Group**	**1998-2000**	**2001**	**2002-2004**	**2005-2007**	**2008-2009**
**A**	**• ** AIDS-defining condition	**• ** AIDS-defining condition	**• ** AIDS-defining condition	**• ** AIDS-defining condition	**• ** AIDS-defining condition
	**• ** Severe symptoms	**• ** Severe symptoms	**• ** Severe symptoms	**• ** Severe symptoms	**• ** Severe symptoms
		**• ** CD4^+^ <200	**• ** CD4^+^ <200	**• ** CD4^+^ <200	**• ** CD4^+^ <200
**B**	**• ** CD4^+^ <500	**• ** CD4^+^ >200 but ≤350	**• ** CD4^+^ >200 but ≤350	**• ** CD4^+^ >200 but ≤350	**• ** CD4^+^ >200 but ≤350
	**• ** VL >10,000 (bDNA) or >20,000 (RT-PCR)				
**C**	**• ** Elective decision	**• ** VL >30,000 (bDNA) or >55,000 (RT-PCR)	**• ** VL >55,000 (bDNA or RT-PCR)	**• ** VL >100,000 (RT-PCR)	**• ** CD4^+^ >350
		**• ** CD4^+^ >350	**• ** CD4^+^ >350	**• ** CD4^+^ >350	**• ** Other elective decision
		**• ** Other elective decision	**• ** Other elective decision	**• ** Other elective decision	

^1^Guideline statements of “some would treat” were interpreted as indicating the elective nature of this decision. Therefore viral load was not included as a Group B criterion after 2000.

The different initiator/delayer classification timeframes for Groups A and B were selected based on practice patterns unique to the military, specifically that some participants do not live near an MTF offering specialty HIV care and receive care on an approximately four to six month cycle. Participants could be evaluated in more than one group if they later met criteria for inclusion in another group, up until the time when they initiated HAART, at which point they could no longer be analyzed in any other group.

To further assess for evidence of disparity, the analysis was repeated evaluating the association between study factors and starting primary pneumocystis pneumonia (PCP) prophylaxis within four months of the first verified CD4 count <200 cells/mm^3^ (i.e. at least one subsequent CD4 count within three months that was also <200 cells/mm^3^). This analysis included those without a history of PCP who met CD4 criteria for primary PCP prophylaxis and had not yet started this treatment. Initiators started PCP prophylaxis within four months and delayers did not.

### Data analysis

Factors studied included demographic characteristics (age, gender, self-reported race/ethnicity, duty status, and rank), HIV-specific factors (time since HIV infection, CD4 cell counts, HIV RNA levels, history of prior AIDS-defining condition and history of HIV symptoms), comorbid medical conditions (cerebrovascular disease, chronic kidney disease, chronic obstructive pulmonary disease, chronic viral hepatitis, cirrhosis, coronary artery disease, diabetes, and malignancy other than basal cell carcinoma) and history of a mental health disorder (anxiety disorder, bipolar disorder, major depression, obsessive-compulsive disorder, post-traumatic stress disorder, schizophrenia, substance abuse, and prior suicide attempt).

Descriptive statistics are presented as means with standard deviations (SD) or medians with interquartile ranges (IQR). For Groups A and B, factors were compared as of the date the participant was categorized into the group. For Group C, comparisons used the date of HAART initiation for initiators and date of last visit for delayers. Within each group, study variables among participants who initiated vs. delayed HAART were compared using logistic regression. Analyses were repeated among participants who initiated vs. delayed primary PCP prophylaxis when indicated. All multivariate models adjusted for gender, age, race/ethnicity, marital status, rank, duty status, military site, branch of service, duration of HIV disease, prior ART use, prior comorbid illnesses, and history of mental health diagnosis. The Group A analyses additionally adjusted for HIV RNA and reason for classification (CD4 cell count <200 cells/mm^3^, AIDS-defining condition, or severe symptoms). The Group C analyses additionally adjusted for HIV RNA and CD4 cell count. To account for changes in recommendations, models for the analyses were stratified by HIV treatment guideline era. Odds ratios are given with 95% confidence intervals (CI). All p-values are two-sided. Analyses were conducted using SAS (version 9.1, Cary, NC).

## Results

We evaluated 1262 participants who were predominantly male (94%) with a mean age of 33.8 years (SD 8.7). Forty percent of participants were Caucasian, 46% African American, 10% Hispanic and 5% other ethnic groups. Sixty-two participants were evaluated in both Group A and Group B.

Of the 1262 participants, 208 were categorized as having a definite indication to start HAART (Group A), including 99 (48%) due to a CD4 count below 200 cells/mm^3^, 99 (48%) due to severe symptoms, and 10 (5%) for an AIDS-defining condition. Of these, 121 (58%) initiated HAART within 4 months of indication, with a median of 11 days (IQR 3-36) from indication to HAART initiation. The remaining 87 (42%) were defined as delayers; reasons for delay were not captured in the study database. The median CD4 cell count was 176 (IQR 131 – 207) among initiators and 417 (IQR 267-554) among delayers.

In both the univariate (UV) and multivariate (MV) models, race/ethnicity and other demographic variables were not significantly associated with the timing of HAART in Group A. Participants with higher HIV RNA levels were more likely to initiate HAART (OR 2.19, 95% CI 1.32-3.64 per 1-log_10_ increase). Participants classified into Group A due to severe symptoms were less likely to initiate HAART when compared to those classified because their CD4 cell count fell below 200 cells/mm^3^ (OR = 0.07, 95% CI 0.03-0.17). There was no significant difference between participants classified into Group A for an AIDS-defining condition when compared with those classified for a CD4 cell count <200 cells/mm^3^ (OR = 0.36, 95% CI 0.07 – 1.93) (Table 2).

**Table 2:** Factors associated with the initiation of HAART from 1998-2009 among those with a definite indication to start (Group A)

			**Univariate models**		Multivariate model*	
	**Initiators (N = 121) N (%) or median (IQR)**	**Delayers (N = 87) N (%) or median (IQR)**	**OR (95% CI)**	**P**	**OR (95% CI)**	**P**
**Demographics**						
Gender (% Male)	107 (88%)	77 (89%)	0.99 (0.42 - 2.35)	0.99	0.55 (0.11 - 2.61)	0.45
Age	35.1 ± 9.4	33.9 ± 9.7	1.01 (0.98 - 1.04)	0.35	1.03 (0.98 - 1.08)	0.26
Race/ethnicity						
Caucasian	41 (33.9%)	35 (40.2%)	Referent		Referent	
American	53 (43.8%)	41 (47.1%)	1.10 (0.60 - 2.03)	0.75	0.69 (0.28 - 1.71)	0.43
Hispanic/Latino/other	27 (22.3%)	11 (12.6%)	2.09 (0.91 - 4.82)	0.08	1.71 (0.53 - 5.50)	0.37
Marital status (% married)	45 (38.5%)	27 (32.5%)	1.30 (0.72 - 2.34)	0.39	1.08 (0.46 - 2.56)	0.86
**Military characteristics**						
Rank						
Officer/warrant	8 (6.6%)	8 (9.2%)	0.70 (0.25 - 1.95)	0.50	0.34 (0.08 - 1.49)	0.15
Enlisted	100 (82.6%)	70 (80.5%)	Referent		Referent	
N/A	13 (10.7%)	9 (10.3%)	1.01 (0.41 - 2.49)	0.98	0.73 (0.08 - 6.73)	0.78
Duty status						
Active	83 (68.6%)	66 (75.9%)	Referent		Referent	
Retired	22 (18.2%)	14 (16.1%)	1.23 (0.59 - 2.60)	0.58	1.34 (0.35 - 5.19)	0.67
Dependent	15 (12.4%)	7 (8.0%)	1.68 (0.65 - 4.37)	0.28	2.56 (0.29 - 22.80)	0.40
**HIV characteristics**						
Duration of HIV infection (years)	1.3 (0.1 - 4.8)	1.9 (0.2 - 7.1)	0.95 (0.90 - 1.02)	0.14	0.92 (0.83 - 1.03)	0.15
Log_10_ HIV RNA	4.7 (4.3 - 5.2)	4.3 (3.7 - 4.8)	2.10 (1.46 - 3.04)	<**0.001**	2.19 (1.32 - 3.64)	**0.002**
CD4 count in cells/mm^3^	176.0 (131.0 - 207.0)	417.0 (267.0 - 554.0)				
Prior ART (non-HAART) use	18 (15%)	13 (15%)	0.99 (0.46 - 2.16)	0.99	0.97 (0.26 - 3.55)	0.96
Prior comorbid illness(es)	10 (8%)	10 (11%)	0.69 (0.28 - 1.75)	0.44	0.30 (0.08 - 1.14)	0.08
History of mental health diagnosis						
Depression	11 (9%)	9 (10%)	0.87 (0.34 - 2.21)	0.77	0.65 (0.18 - 2.42)	0.52
Other mental health diagnosis	9 (7%)	6 (7%)	1.07 (0.36 - 3.14)	0.90	1.13 (0.27 - 4.83)	0.86
None	101 (83%)	72 (83%)	Referent		Referent	
Reason for classification						
Severe symptoms	34 (28%)	65 (75%)	0.12 (0.06 - 0.22)	<**0.001**	0.07 (0.03 - 0.17)	<**0.001**
AIDS defining condition	6 (5%)	4 (5%)	0.33 (0.09 - 1.30)	0.11	0.36 (0.07 - 1.93)	0.23
CD4 count <200 cells/mm^3^	81 (67%)	18 (21%)	Referent		Referent	

*Multivariate model additionally adjusts for branch and site of service.

P-values < 0.05 are presented in bold text.

Group B included 637 participants, with 248 (39%) initiators and 389 (61%) delayers. Of these, 476 (75%) were classified into this group because of CD4 count, 82 (13%) due to HIV RNA level, and 79 (12%) because of meeting a combination of these factors. As in Group A, race/ethnicity was not significantly associated with the timing of HAART in Group B in either model. Factors associated with initiating HAART in both the UV and MV models were duration of HIV infection (OR 0.87, 95% CI 0.0.80-0.94 per 1-year increase) and prior non-HAART antiretroviral therapy (ART) use (OR 0.51, 95% CI 0.27-0.97). Additional factors that were significant only in univariate analyses were age, military status, and comorbid illnesses (Table 3).

**Table 3:** Factors associated with the initiation of HAART from 1998-2009 among those classified into the consider offering treatment group (Group B)

			**Univariate models**		Multivariate model*^,^**	
	**Initiators (N = 248) N (%) or median (IQR)**	**Delayers (N = 389) N (%) or median (IQR)**	**OR (95% CI)**	**p**	**OR (95% CI)**	**p**
**Demographics**						
Gender (% Male)	236 (95%)	368 (95%)	1.04 (0.50 - 2.16)	0.92	1.19 (0.44 - 3.22)	0.74
Age	31.7 ± 7.3	33.1 ± 8.4	0.98 (0.96 - 1.00)	**0.04**	1.00 (0.97 - 1.03)	0.93
Race/ethnicity						
Caucasian	102 (41.1%)	141 (36.2%)	Referent		Referent	
African American	109 (44.0%)	195 (50.1%)	0.80 (0.56 - 1.13)	0.20	0.83 (0.56 - 1.24)	0.37
Hispanic/Latino/other	37 (14.9%)	53 (13.6%)	0.91 (0.55 - 1.49)	0.71	1.05 (0.60 - 1.82)	0.88
Marital status (% married)	83 (34.6%)	120 (33.9%)	1.05 (0.74 - 1.49)	0.78	1.10 (0.75 - 1.62)	0.62
**Military characteristics**						
Rank						
Officer/warrant	19 (7.7%)	28 (7.2%)	1.01 (0.55 - 1.86)	0.97	1.14 (0.56 - 2.32)	0.72
Enlisted	214 (86.3%)	334 (85.9%)	Referent		Referent	
N/A	15 (6.0%)	27 (6.9%)	0.95 (0.49 - 1.84)	0.88	0.97 (0.20 - 4.77)	0.97
Duty status						
Active	219 (88.3%)	297 (76.3%)	Referent		Referent	
Retired	17 (6.9%)	77 (19.8%)	0.34 (0.19 - 0.59)	<**0.001**	0.61 (0.28 - 1.32)	0.21
Dependent	12 (4.8%)	15 (3.9%)	1.15 (0.53 - 2.52)	0.72	2.20 (0.36 - 13.35)	0.39
**HIV characteristics**						
Duration of HIV infection (years)	0.3 (0.1 - 1.9)	1.8 (0.2 - 5.2)	0.84 (0.78 - 0.89)	<**0.001**	0.87 (0.80 - 0.94)	<**0.001**
Log_10_ HIV RNA	4.5 (4.1 - 4.9)	4.1 (3.4 - 4.5)				
CD4 cell count	323.0 (287.0 - 422.5)	352.0 (316.0 - 461.0)				
Prior ART (non-HAART) use	22 (9%)	100 (26%)	0.30 (0.18 - 0.51)	<**0.001**	0.51 (0.27 - 0.97)	**0.04**
Prior comorbid Illness (es)	12 (5%)	40 (10%)	0.45 (0.23 - 0.89)	**0.02**	0.76 (0.35 - 1.66)	0.49
History of mental health diagnosis						
Depression	18 (7%)	37 (10%)	0.73 (0.40 - 1.32)	0.30	1.02 (0.53 - 1.98)	0.95
Other mental health diagnosis	13 (5%)	20 (5%)	0.96 (0.47 - 1.99)	0.92	1.16 (0.53 - 2.55)	0.71
None	217 (88%)	332 (85%)	Referent		Referent	
Reason for classification						
CD4 <500 (1998-2000) CD4 <350 (2001-2008)	168 (68%)	308 (79%)				
HIV RNA >20,000 copies/ml (1998-2000 only)	32 (13%)	50 (13%)				
CD4 <500 and HIV >20,000 copies/ml (1998-2000 only)	48 (19%)	31 (8%)				

*All models are stratified by guideline era (1998-2000, 2001-2009).

**Multivariate model additionally adjusts for branch and site of service.

P-values < 0.05 are presented in bold text.

Among the 479 participants in Group C, 144 (30%) participants initiated HAART. In UV models, African Americans were half as likely to initiate HAART in this group when compared to Caucasians (OR 0.50, 95% CI 0.32-0.78). In the MV model, African American race remained significantly associated with delayed HAART initiation (OR 0.49, 95% CI 0.25-0.97) (Table 4).

**Table 4:** Factors associated with the elective initiation of HAART from 1998-2009, Group C

			**Univariate models**		Multivariate model*^,^**	
	**Initiators (N = 144) N (%) or median (IQR)**	**Delayers (N = 335) N (%) or median (IQR)**	**OR (95% CI)**	**p**	**OR (95% CI)**	**P**
**Demographics**						
Gender (% Male)	140 (97%)	310 (93%)	3.19 (1.08 - 9.47)	**0.04**	1.50 (0.34 - 6.63)	0.59
Age	33.6 ± 9.4	32.2 ± 8.5	1.01 (0.99 - 1.04)	0.20	1.04 (0.99 - 1.09)	0.08
Race/ethnicity						
Caucasian	70 (48.6%)	117 (34.9%)	Referent		Referent	
African American	47 (32.6%)	162 (48.4%)	0.50 (0.32 - 0.78)	**0.002**	0.49 (0.25 - 0.97)	**0.04**
Hispanic/Latino/other	27 (18.8%)	56 (16.7%)	0.88 (0.51 - 1.53)	0.66	0.85 (0.40 - 1.82)	0.68
Marital status (% married)	38 (27.7%)	95 (31.8%)	0.67 (0.41 - 1.08)	0.10	0.67 (0.34 - 1.33)	0.25
**Military characteristics**						
Rank						
Officer/warrant	23 (16.0%)	30 (9.0%)	1.79 (0.99 - 3.24)	**0.05**	1.30 (0.56 - 3.04)	0.54
Enlisted	116 (80.6%)	275 (82.1%)	Referent		Referent	
N/A	5 (3.5%)	30 (9.0%)	0.27 (0.10 - 0.74)	**0.01**	1.53 (0.19 - 12.62)	0.69
Duty status						
Active	121 (84.0%)	284 (84.8%)	Referent		Referent	
Retired	18 (12.5%)	32 (9.6%)	1.09 (0.58 - 2.06)	0.78	0.52 (0.13 - 2.13)	0.36
Dependent	5 (3.5%)	19 (5.7%)	0.50 (0.18 - 1.39)	0.18	0.38 (0.04 - 3.52)	0.40
**HIV characteristics**						
Duration of HIV Infection (years)	1.2 (0.3 - 4.0)	2.1 (0.7 - 4.8)	0.92 (0.86 - 0.98)	**0.009**	0.91 (0.80 - 1.04)	0.18
Log_10_ HIV RNA^+^	4.5 (3.7 - 5.0)	3.7 (3.0 - 4.4)	3.11 (2.30 - 4.21)	<**0.001**	3.25 (2.15 - 4.93)	<**0.001**
CD4 cell count***	496.5 (398.5 - 627.5)	606.0 (496.0 - 775.0)	0.83 (0.78 - 0.89)	<**0.001**	0.82 (0.75 - 0.90)	<**0.001**
Prior ART (non-HAART) use	29 (20%)	19 (6%)	3.49 (1.82 - 6.71)	<**0.001**	50.56 (9.94 - 257.1)	<**0.001**
Prior comorbid illness (es)	12 (8%)	21 (6%)	1.32 (0.62 - 2.77)	0.47	0.51 (0.16 - 1.62)	0.25
History of mental health diagnosis						
Depression	14 (10%)	43 (13%)	0.74 (0.39 - 1.41)	0.36	0.44 (0.17 - 1.10)	0.08
Other mental health diagnosis	8 (6%)	35 (10%)	0.52 (0.23 - 1.15)	0.11	0.37 (0.13 - 1.04)	0.06
None	122 (85%)	257 (77%)	Referent		Referent	

*All models are stratified by guideline era (1998-2000, 2001-2009).

**Multivariate model additionally adjusts for branch and site of service.

^+^Per 1.0 log_10_ increase.

***Univariate and Multivariate models are per 50 cells/mm^3^.

P-values < 0.05 are presented in bold text.

There were 408 participants with a CD4 count <200 cells/mm^3^ included in the analysis of factors associated with the initiation of primary PCP prophylaxis. Race/ethnicity, age, and gender were not associated with the timing of primary PCP prophylaxis. HAART-experienced participants were less likely to start PCP prophylaxis (OR 0.48, 95% CI 0.25-0.91, compared to ART-naive participants) as were those with CD4 cell counts closer to 200 cells/mm^3^ (OR 0.55, 95% CI 0.42 – 0.70 per 50 cells/mm^3^) (Table 5).

**Table 5:** Factors associated with early initiation of primary PCP prophylaxis from 1998-2009

			**Univariate models**		Multivariate model*	
	**Initiators (N = 156) N (%) or median (IQR)**	**Delayers (N = 252) N (%) or median (IQR)**	**OR (95% CI)**	**p**	**OR (95% CI)**	**P**
**Demographics**						
Gender (% Male)	144 (92%)	227 (90%)	1.32 (0.64 - 2.71)	0.45	1.00 (0.33 - 3.03)	>0.99
Age	36.9 (29.2 - 43.0)	37.4 (32.2 - 42.5)	0.99 (0.97 - 1.01)	0.36	0.98 (0.95 - 1.01)	0.27
Race/ethnicity						
Caucasian	61 (39%)	100 (40%)	Referent		Referent	
African American	72 (46%)	122 (48%)	0.97 (0.63 - 1.49)	0.88	1.21 (0.71 - 2.09)	0.48
Hispanic/Latino/other	23 (15%)	30 (12%)	1.26 (0.67 - 2.36)	0.48	1.35 (0.61 - 3.00)	0.46
Marital status (% married)	63 (41%)	71 (31%)	1.50 (0.98 - 2.30)	0.06	1.52 (0.89 - 2.59)	0.12
**Military characteristics**						
Rank						
Officer/warrant	19 (12%)	24 (10%)	1.33 (0.70 - 2.54)	0.38	1.76 (0.74 - 4.15)	0.20
Enlisted	121 (78%)	199 (79%)	Referent		Referent	
N/A	16 (10%)	24 (10%)	1.12 (0.57 - 2.20)	0.73	1.72 (0.54 - 5.47)	0.36
Duty status						
Active	82 (53%)	109 (43%)	Referent		Referent	
Retired	63 (40%)	118 (47%)	0.71 (0.47 - 1.08)	0.11	1.16 (0.59 - 2.25)	0.67
Dependent	11 (7%)	25 (10%)	0.58 (0.27 - 1.26)	0.17	0.36 (0.09 - 1.53)	0.17
**HIV characteristics**						
Duration of HIV infection (years)	2.5 (0.1 - 8.8)	6.6 (1.8 - 10.9)	0.93 (0.89 - 0.97)	<**0.001**	0.99 (0.93 - 1.05)	0.70
Log_10_ HIV RNA	4.8 (4.3 - 5.2)	4.5 (3.6 - 5.0)	1.49 (1.21 - 1.83)	<**0.001**	1.25 (0.98 - 1.60)	0.07
CD4 count*	130.0 (68.5 - 167.0)	169.0 (131.5 - 187.0)	0.59 (0.49 – 0.72)	<**0.001**	0.55 (0.42 – 0.70)	<**0.001**
Prior ART use						
None	92 (59%)	81 (32%)	Referent		Referent	
ART	14 (9%)	24 (10%)	0.51 (0.25 - 1.06)	0.07	0.81 (0.30 - 2.15)	0.67
HAART	50 (32%)	147 (58%)	0.30 (0.19 - 0.46)	<**0.001**	0.48 (0.25 - 0.91)	**0.03**
Prior AIDS	24 (15%)	36 (14%)	1.09 (0.62 - 1.91)	0.76	1.00 (0.45 - 2.19)	>0.99
History of mental health diagnosis						
Depression	19 (12%)	46 (18%)	0.63 (0.35 - 1.13)	0.12	0.82 (0.41 - 1.65)	0.58
Other mental health diagnosis	13 (8%)	16 (6%)	1.24 (0.58 - 2.68)	0.58	1.52 (0.61 - 3.79)	0.36
None	124 (79%)	190 (75%)	Referent		Referent	

*Univariate and Multivariate models are per 50 cells/mm^3^.

## Discussion

The interplay of factors surrounding the timing of HAART initiation is complex, and involves not only biologic factors such as immune function and disease progression, but also psychosocial and systems-based factors. A better understanding of these factors may help eliminate treatment barriers and enable healthcare providers to offer optimal care to all patients with HIV. This study’s findings show that in an environment providing no-fee open access care, certain factors previously demonstrated to affect the timing of HAART, namely race/ethnicity, age, gender and depression did not have the same impact in most patients in this cohort. This remained true with respect to timely initiation of primary PCP prophylaxis when indicated. However in the subset of patients in Group C who electively started HAART at higher CD4 cell counts without an indication, African American race/ethnicity was associated with lower odds of initiation.

The Department of Defense provides a healthcare system ensuring access to visits and medications for its beneficiaries. In addition, keeping appointments is reinforced for active duty members by their chain of command. This practice and the military’s culture of health provide a potential explanation for the absence of a statistically significant difference in the timing of HAART initiation when indicated across demographic groups in this cohort. However the potential impact of command-directed follow-up among active duty personnel may be suggested by the observation of a non-significant trend towards lower odds of HAART initiation among retired participants. Previous studies have underscored the importance of regular clinic follow-up on appropriate HAART use. In one study which showed a longer time to HAART initiation among black patients, the most common reasons for delays in referral to case conferences (where decisions were made about HAART) were lack of regular follow-up, active substance use, and the patient’s desire to not use HAART [21]. There are fewer issues with access and follow-up in the U.S. military healthcare system. Also, the DoD Drug Demand Reduction Program with random urine drug screening has been very successful in decreasing substance abuse among active duty, especially with regard to intravenous drug use [22]. Cultural differences in medication uptake and adherence have yet to be fully explored in the military setting, but preliminary evaluation suggests HAART adherence levels are equally high among a subset of African Americans and Caucasians [18].

African Americans in this study were less likely than Caucasians to electively start HAART at higher CD4 counts. And while there were no statistically significant ethnic differences among those with an indication for HAART, there remained a trend towards lower odds of HAART initiation at lower CD4 count thresholds among African American participants when compared with Caucasian participants. A number of previous studies observed disparities in HAART use among certain race/ethnic groups and women although these did not specifically evaluate the elective initiation of HAART at high CD4 counts [8-10,14,21,23-26]. While the etiology of these disparities is largely attributed to differences in healthcare access, other factors have also been shown to contribute and perhaps offer an explanation for the findings in this study. For example, racial discordance of the physician-patient relationship may be associated with cultural miscommunication that could explain delays in receipt of HAART [12,27]. Additionally, patients who reported that their provider “knows me as a person” were more likely to receive HAART than those who did not report this belief [13]. Other factors may also impact decisions about HAART including health literacy and attitudes toward health [28,29]. It is important to note that not all studies have shown racial/ethnic differences in the receipt of HAART. In a study performed among HIV-positive patients in Denmark, where healthcare is free, both white and nonwhite patients had similar rates of receiving HAART, and race/ethnicity did not have an apparent effect on the outcomes associated with HAART [30].

The implication of the ethnic difference in elective HAART initiation in this study is important because some studies have demonstrated a differential response to HAART between Caucasian and non-Caucasian groups [16,17,21,31,32]. In an analysis of antiretroviral-naïve patients with HIV who started HAART between 1997 and 2003, African American race/ethnicity was associated with failure to reach virologic suppression at 12 months [16] and at 6 months and 12 months after initiating HAART [17]. And despite continuous use of HAART, black women experienced increased rates of AIDS-defining illness and death from AIDS when compared with white women [32]. Interestingly, two studies suggest that in spite of decreased rates of virologic suppression, African Americans and Caucasians treated with HAART in the DoD have equivalent long-term outcomes [15,18]. Pharmacogenomic and adherence differences are being explored as possible explanations for the lower virologic suppression rates.

Depression was not associated with the timing of HAART in this study among those with an indication to start, although it has been shown to impact the decision to start HAART in some previous studies [33,34] but not all [35]. There was a delay in HAART initiation in Group A due to the presence of severe symptoms, which may reflect the high median CD4 cell count among delayers in this group, a prioritization of disease management, or possibly a concern about immune reconstitution inflammatory syndrome (IRIS) among those with lower nadir CD4 cell counts. Prior mono- and dual-NRTI use was associated with decreased odds of starting HAART in Group B, but increased odds in Group C. This effect was highly associated with calendar year and may reflect delays in changing to HAART in the early HAART era in participants stable on non-HAART ART in Group B versus a preference for newer therapies in those electing to start HAART in Group C. A similar phenomenon in the early HAART era is suggested in a study examining the reasons why 28 of 60 patients treated for HIV in a U.S. health clinic were not treated with HAART in 1997-1998 in accordance with DHHS guidelines at the time [36]. Another study also suggested delays in transitioning to the use of protease inhibitors in a U.S. practice in 1996 among 190 patients; higher CD4 cell count (200-500 cells/mm^3^) was a prominent factor in multivariate models [37].

There are some study limitations. It is a retrospective analysis covering a broad timeframe and involves five DoD research sites among which practice patterns may vary. Statistical models were adjusted to account for site of visit and service affiliation. While the cohort has a balance of male African American and Caucasian participants, other ethnic groups and women are represented in much smaller proportions, making it difficult to draw conclusions about the timing of HAART among these groups. Overall, the cohort is relatively small, and while we observed many trends that did not reach statistical significance, it is possible that these trends would represent clinically important differences in a larger cohort. Further, it is likely that there are additional confounding factors that we were not able to fully account for in the analysis. The criteria for determining eligibility for HAART initiation in this study are limited to the DHHS guidelines, even though other societies in the U.S. and worldwide have published guidelines that may have influenced practice patterns. And importantly, there are limited data on substance use in this cohort, although it is estimated to be quite low, with no IVDU reported at last survey [22].

## Conclusion

In a cohort with free access to HIV care and medications, race/ethnicity, age, and gender did not impact HAART timing among those with an indication for therapy, but African American race/ethnicity was associated with lower odds of electively initiating antiretrovirals. This study reinforces that free healthcare can potentially overcome some of the observed disparities in HIV care, particularly those related to access. However additional unmeasured factors may result in persistent differences in elective care decisions.

## Competing interests

The authors declare that they have no competing interests.

## Authors’ contributions

ENJ, MPR, and BKA designed and conducted the study, interpreted the data, and prepared and edited the manuscript. MLL, NFC, ACW, AG, JFO, and GEM assisted with study design, data interpretation, and editing the manuscript. All authors read and approved the final manuscript.
